# Multi-Response Optimisation of Thermal Barrier Coating Performance Based on Grey-Based Fuzzy Approach

**DOI:** 10.3390/ma19061110

**Published:** 2026-03-12

**Authors:** Zhe Zou, Mingder Jean

**Affiliations:** College of Arts and Design, Jimei University, 185 Yinjiang Rd., Jimei District, Xiamen 361021, China; zouzhe@jmu.edu.cn

**Keywords:** zirconia, plasma spraying, grey-based fuzzy, coatings and surface properties

## Abstract

This study applies grey theory alongside fuzzy models based on Taguchi design experiments to optimise the performance of coatings for multiple responses, which enhances the quality of plasma-sprayed ceramic coatings. An L18 orthogonal array experiment with eight control factors was conducted, and the impact of the control parameters on the surface properties of the coatings was critically evaluated. In addition, an analysis of variance was conducted, and the surface structure of the coatings was examined using SEM. The multi-response characteristics of surface roughness, porosity, hardness, coating thickness and wear depth values during the spraying of ceramic coatings were studied comparatively through optimisation. In addition, a confirmation experiment was conducted. The experimental results show that surface roughness was reduced by 15.96%, porosity by 65.35%, hardness by 34.60%, wear depth by 34.04%, and coating thickness by 32.01% through optimal factors by plasma spraying coatings. Overall, a 48.94% improvement in multi-properties was observed when compared to the initial settings. Based on the above results, this study employed Taguchi methods to optimize the modeling of plasma spraying using grey-based fuzzy theory, thereby significantly enhancing the multi-response quality of plasma-sprayed coatings. The expected outcomes in terms of coating surface properties have also been achieved by these results.

## 1. Introduction

Thermal spraying technology has attracted a lot of attention as a process for applying protective coatings. It is an exceptionally promising and cost-effective solution because it significantly improves the properties of processed materials while requiring only a small amount of powder [1,2,3,4]. The different types of thermal spraying processes are categorised by the heat source and the material form. There are several methods involved in thermal spraying technology, including flame spraying, arc spraying and plasma spraying [5,6,7,8]. Plasma spraying uses a plasma arc as its heat source, which can reach temperatures in excess of 10,000 °C to melt high-melting-point materials such as ceramics and cermets. The resulting dense coatings exhibit superior properties, which make them suitable for high-end applications such as aerospace engine components and mould reinforcement [9,10]. Unlike other thermal spraying processes, which are typically limited to low-melting-point metals or alloys, plasma spraying reliably melts ceramics, cermets, and high-melting-point metals. The projected particles are fully molten and uniformly atomised, forming dense coatings with significantly higher bond strengths than those achieved by traditional flame or arc spraying processes [11,12]. It can accommodate a variety of materials and component geometries. Conversely, significant material limitations are imposed by high-speed oxy-fuel spraying, while complex equipment is required by explosive spraying, which is only applicable to large components with simple structures. Meanwhile, the original properties of the substrate are fully preserved due to the extremely minimal heat-affected zone. In contrast, explosive spraying induces severe thermal shock, while conventional flame spraying causes extensive thermal diffusion. These characteristics of plasma-sprayed coatings significantly enhance their wear and corrosion resistance, and they demonstrate superior performance in high-temperature environments.

In recent years, sprayed ceramic and cermet coatings (such as ZrO_2_, Al_2_O, Cr_2_O_3_ and WC/Co) have received a lot of attention because they can solve many industrial problems [13,14]. Yttria-stabilised zirconia (ZrO_2_/8% Y_2_O_3_) ceramics are primarily used for hard, wear-resistant coatings, for which surface properties are critical. Numerous studies have demonstrated the promising properties of plasma-sprayed zirconia ceramic coatings. Gell et al. [15] revealed that a sol–gel process was employed to synthesise a hybrid coating. The study revealed that the hybrid coating exhibits a dense structure, effectively impeding the penetration of corrosive media. Praveen et al. [16] report on the preparation and high-temperature performance of yttria-stabilised zirconia (YSZ) thermal barrier coatings (TBCs) on carbon fibre-reinforced polymer (CFRP) composites. The results showed that the YAG TBC, with a precisely controlled pore volume of around 35%, had a thermal conductivity of just 0.57–0.64 W/m·K at temperatures between 25 and 400 °C, which is significantly lower than that of conventional dense YAG materials. Goudarzi et al. [17] conducted a comparative study investigating the thermal–mechanical properties of functionally graded thick thermal barrier coatings with varying composition and porosity gradients. Key findings indicate that gradually matching the thermal expansion coefficient by continuously varying the material composition reduces the risk of delamination, while also effectively mitigating thermal stresses and enhancing thermal shock resistance. Lv et al. [18] proposed that synergy resistance could be enhanced by designing thermal barrier coating systems with a gradient pore distribution. Yttria-stabilised zirconia coatings with different pore structures were produced by altering the parameters of the APS process. The deposition of CoCrFeNiMo_0.2_ high-entropy alloy powder onto a 316L stainless steel substrate was carried out using high-velocity air-fuel spraying technology, as reported by Ye et al. [19]. The coating is about 1.8 times harder than the substrate, and the strength of the bond between the coating and substrate is increased to 39.4 MPa. Among these, the existing literature on plasma spray coatings primarily focuses on coatings that are highly resistant to wear, enhancing the microstructure and mechanical properties of substrate surfaces. Research efforts concentrate on addressing issues such as residual stresses, porosity, cracking, deformation, and delamination [20,21,22,23]. It has been confirmed by the aforementioned findings that excellent protective properties have been demonstrated by plasma spray coatings in solving the issues faced by surface treatments on substrates [24,25]. There is often a simultaneous occurrence of multiple noteworthy response phenomena as a result of the trend towards increasingly diversified manufacturing. These responses are all subject to the influence of various variables, which necessitates synchronous analysis. Moreover, the detailed account of numerical solutions for the properties of deposits during the spraying process is extremely rare in relevant studies [26,27,28], whilst the welding engineering field still lacks practical guidelines for well-established models. Alternatively, it is anticipated that alternative strategies will emerge to address the multiple response issues associated with plasma-sprayed deposits.

In recent years, several studies have been conducted on the multi-response properties of plasma-sprayed coatings. The conventional approach to mathematical modelling and analysis in plasma spraying processes is of the essence, particularly in the domain of accelerated industrial production. This process involves complex interactions among multiple parameters that directly affect coating quality [29,30,31]. Due to the complex nature of plasma-spraying manufacturing systems, the interactions between operating parameters, coupled with the presence of numerous uncertainties in the manufacturing environment, mathematical equations alone often prove insufficient for the resolution of these issues. This results in the model for the process being extremely complex, thereby making it difficult to implement. This paper addresses three core challenges in plasma spraying technology: the multi-parameter coupling, the inability of traditional methods to achieve synergistic optimisation of multiple coating properties, and the lack of systematic optimisation models in existing research. To address these challenges, the literature employs various artificial intelligence techniques to model systems and evaluate the performance of developed systems [32,33]. These techniques include fuzzy logic, grey systems, and adaptive neural networks, which can model input/output relationships and process parameters while simultaneously enhancing the quality characteristics of both products and manufacturing processes [34,35,36,37].

Most studies on plasma-sprayed coatings use single-response analysis rather than multi-response analysis, which is currently done by considering each response variable separately. This approach fails to capture the full spectrum of sprayed coating performance. Additionally, a more significant challenge emerges from the fact that multi-response metrics are essentially combinations of weighted individual objectives [38,39,40]. However, rather than using mathematical analysis to study the characteristics of uncertain systems, grey system theory uses grey relational analysis to address multi-objective behaviour [41]. The relationships between the various properties and parameters in the plasma spraying process are nonlinear. As nonlinearity issues cannot be resolved by grey systems, limitations in linear problems are addressed by employing fuzzy logic reasoning. There is potential for it to resolve weighting challenges in multi-objective problems [42,43,44,45,46,47].

The aim of this study is to enhance multi-response solutions through the integration of grey systems and fuzzy logic analysis, underpinned by Taguchi design. Meanwhile, the spray-coating process for zirconia coatings with multiple response characteristics was optimised [48,49,50,51,52,53], and the influence of spray parameters on coating quality was investigated, thereby contributing to enhancing the mechanical properties of the coatings [54,55]. The mechanical properties include surface roughness, porosity percentage, Vickers hardness, wear depth and coating thickness [56,57,58,59,60,61]. The unresolved problem of weighting each response attribute for multiple qualities is addressed by utilising the inference rule database in fuzzy logic, coupled with calculation through the grey system. This methodology combines grey system analysis and fuzzy logic, which is expected to improve the performance of plasma-spraying coatings.

## 2. Experiments

### Materials and Preparations

**Figure 1:**
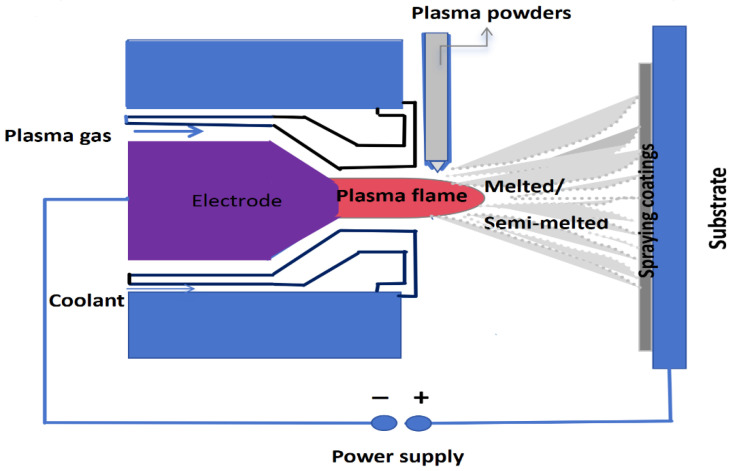
Schematic of a plasma spraying system comprising a gun electrode with a nozzle or substrate, ionised argon gas, arc-melted/semi-melted powder, and the resulting coating.

The underlying principle is illustrated in Figure 1. A high voltage is applied between the gun electrode and the nozzle or substrate, where a high-frequency oscillator activates the gas (argon, nitrogen, hydrogen, etc.) to be ionised, thereby forming an arc. It is then ejected at high velocity onto the workpiece surface, the spreading liquid particles rapidly cool and solidify within microseconds, ultimately forming a flake-like structure. The plasma spraying process was used to prepare Y_2_O_3_-stabilised zirconia coatings (YSZ) in this study. YSZ, a commonly employed thermal spray coating material for thermal insulation and protection, was used as the coating material. This material is characterised by high-temperature resistance, thermal insulation and wear resistance. A special coating powder made by the Sulzer Metco company in Winterthur, Switzerland was chosen, with a particle size of approximately 80–120 μm, and its chemical composition is shown in Table 1. This spraying equipment uses a plasma spraying system with an F4-type plasma gun, which is manufactured by Sulzer Metco. The coating was deposited onto an aluminium-based alloy substrate measuring 150 mm in length, 100 mm in width and 10 mm in thickness. YSZ coatings are often used to change the surface of aluminium-based alloys. This makes them much better at resisting high-temperature corrosion and wear. The porosity is determined using image analysis. This method involves capturing high-resolution images of the coating cross-section using a microscope. The percentage of the total field of view occupied by the porosity area is then calculated using software for image analysis, thereby determining the percentage of porosity. The surface hardness was gauged using a micro Vickers hardness tester (HVS-1000Z, Huazheng Electric Manufacturing (Baoding) Co., Ltd., Huazheng, Baoding, China). The test load was 300 gf, and the dwelling time was 10 s. The structure of the sprayed Y_2_O_3_-stabilised ZrO_2_ coating was analysed using a S-2600H scanning electron microscope (Hitachi, Tokyo, Japan) and an energy-dispersive X-ray spectrometer. The measurement of coating surface roughness was undertaken using a TalyScan 150 (Taylor Hobson, Leicester, UK) surface profiler. Furthermore, a series of wear tests was conducted on the ZrO_2_ coating, which underwent a 100 m test using a ball-on-disc wear instrument. The test uses reciprocating circular motion with ball-to-disc contact, where the disc remains stationary and the ceramic ball slides back and forth along the disc surface. The test parameters were test load—5 N, sliding speed—0.1 m/s, and sliding distance—100 m. A TalyScan 150 (Taylor Hobson, Leicester, UK) surface profiler was employed to measure the depth of the wear tracks. The natural and coded values of the variables are listed in Table 2. It was evaluated through the 18 experiments of the Taguchi design, as depicted in Table 3 by the L18 orthogonal array. The surface properties of the coating were measured through five characteristics, respectively, including surface roughness, porosity, hardness, wear depth, and coating thickness, all of which serve to assess its performance.

**Figure 2:**
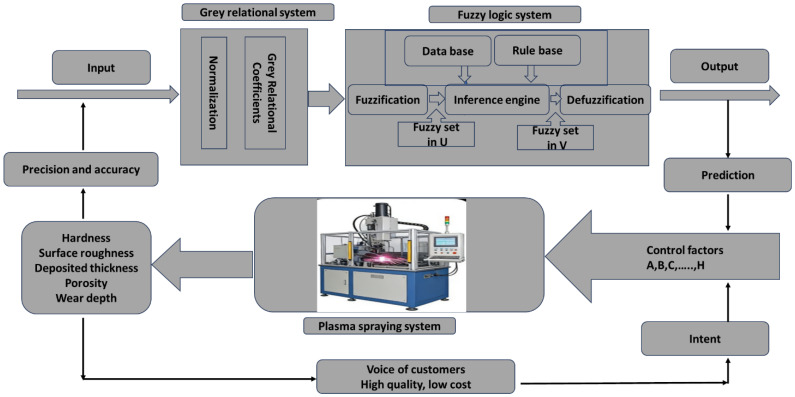
The workflow diagram for plasma spraying coatings developed in this study employs a grey-based fuzzy in conjunction with the Taguchi method.

The intelligent control system for plasma spraying, which is based on a grey-based fuzzy, is presented in Figure 2 as a detailed flowchart. The plasma spraying process can be predicted and optimised through model-based intelligent control, as illustrated by this framework. This flowchart is based on a grey-based fuzzy. This system integrates front-end inputs with a grey relational system. It incorporates the plasma spraying system. It also receives control factors. These include current, voltage, powder feed rate, etc. The quality attributes of the coating are measured after the actual execution of spraying. These attributes include hardness, surface roughness, coating thickness, porosity and wear depth. These quality measures directly meet customer demands for high quality and low cost. A feedback loop is formed to drive continuous system optimisation. Simultaneously, the accuracy of input data is ensured through fuzzy reasoning. This optimises parameters and controls quality during the spraying process. The grey-based fuzzy generates quality predictions, forecasting coating quality under given parameters. The process is first to identify critical parameters using grey relational analysis. Then, it employs fuzzy logic to handle uncertainties. This significantly enhances process stability and coating quality consistency. The closed-loop feedback mechanism automatically adjusts process parameters based on customer requirements and real-time quality inspection results, continuously working towards the objective of high quality and low cost. This system embeds expert experience into the fuzzy logic rule base. This reduces reliance on experienced engineers. It also standardises and reuses process knowledge. In summary, the flowchart of the smart control system for plasma spraying using a grey-based fuzzy shows that this system is very good at dealing with difficult processes like plasma spraying. Plasma spraying is known for having many variables and not having all the information. The system also deals with the complicated non-linear behaviours that happen during the spraying process using fuzzy logic. It replaces traditional mathematical models with fuzzy rules and a knowledge base. This design makes it easy to adjust the spraying settings to get the best results, while also meeting users’ needs for high quality and low cost.

## 3. Experimental Analysis

### 3.1. Control Factors and Their Levels

The robust design, which was introduced by Genichi Taguchi [33], is an effective method for the development of high-quality systems. The Taguchi method offers a particular type of orthogonal array that allows the entire parameter space to be studied with only a fraction of the usual number of experiments. By applying the Taguchi method, which is based on orthogonal arrays, the time and cost required to conduct experiments can be reduced. These arrays provide a set of well-balanced experiments that simultaneously accommodate many design factors and Taguchi’s signal-to-noise ratios (SNRs). The SNRs measure quality with an emphasis on variation and serve as objective functions for optimisation. They also help with data analysis and predicting optimum results. The parameters for this study were determined based on expert experience and literature reports [3,4,5]. Eight controllable operating parameters were experimentally assigned to the left side of the table, including coating layers, acceleration voltage, arc current, travel speed, stand-off distance, powder feed rate, carrier gas, and primary gas. The parameters are detailed in Table 1. Except for coating layers, which had only two levels, all other factors were set at three levels. An orthogonal design with L18 was employed for the experiments. The tested parameters are listed in Table 2 along with their alternative levels used in the experiment. In addition, the experimental results for the hardfacing properties of plasma spray ZrO_2_ coatings, which include surface roughness (μm), porosity percentage (%) and hardness (Hv), worn depth (μm) and deposited thickness (μm) are included in Table 3. In this study, the main objective is to optimise process variables for multi-response in plasma spray coatings. Since the Taguchi method is only applicable to single-response optimisation, the optimisation approach for multi-response differs from that for single-response. Based on the above discussion, this paper employs a grey- based fuzzy approach to optimize multi-response characteristics, grounded in Taguchi design.

### 3.2. The Normalisation of Properties of Coatings

The nature of coating properties dictates several standardized attributes. These depend on the type of performance. There are three types: nominal-is-best, smaller-the-better, and larger-the-better. The larger-the- better attribute includes hardness, the nominal-is-best attribute includes coating thickness, and the -smaller- the better attribute includes surface roughness, hardness, and wear depth. All of these attributes are between 0 and 1, as shown on the right side of Table 3. The larger-the-better attribute can be expressed as:

(1) $${\hat{x}}_{i}\left(k\right)=\frac{x_{i}\left(k\right)-\underset{\forall i}{\min}\;x_{i}\left(k\right)}{\underset{\forall i}{\max}\;x_{i}\left(k\right)-\underset{\forall i}{\min}\;x_{i}\left(k\right)}$$

and the smaller-the-better attribute can be expressed as:

(2) $${\hat{x}}_{i}\left(k\right)=\frac{\underset{\forall i}{\max}\;x_{i}\left(k\right)-x_{i}\left(k\right)}{\underset{\forall i}{\max}\;x_{i}\left(k\right)-\underset{\forall i}{\min}\;x_{i}\left(k\right)}$$

and the nominal-the-better attribute can be expressed as:

(3) $${\bar{x}}_{i}=1-\frac{\left|x_{i}^{\left(0\right)}\left(k\right)-\hat{x}\right|}{max\left\{max\left[x_{i}^{\left(0\right)}\left(k\right)\right]-\hat{x},\hat{x}-min\left[x_{i}^{\left(0\right)}\left(k\right)\right]\right\}}$$

where $x_{i}(k)$, *i* = 1,2,…,18; *k* = 1,2,…,3, is the value for the ith test. The $\underset{\forall i}{\min}\;x_{i}(k)$ is the smallest value $x_{i}(k)$ for the ith test, and $\underset{\forall i}{\max}\;x_{i}(k)$ is the largest value $x_{i}(k)$ for the ith test.

### 3.3. Fuzzy Logic Analysis

**Figure 3:**
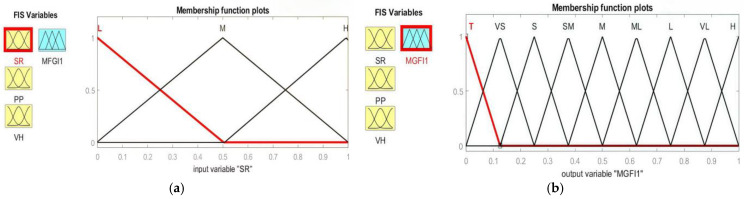
Membership function for the inputs of five grey relational coefficients (**a**) and the output of the multi-grey fuzzy index (**b**).

Fuzzy logic, which is grounded in the mathematical theory of fuzzy sets and extends classical set theory, was proposed by Lotfi Zadeh in 1965 [15]. The concept of degree in condition verification is introduced by fuzzy logic, allowing conditional states to transcend the binary opposition of truth and falsehood. This provides highly valuable flexibility for reasoning, thereby handling imprecision and uncertainty. This technique offers an alternative approach within the field of computing. It is used to represent the linguistic and subjective attributes inherent in variables within the real world. It can be applied to control systems and other domains, enhancing the efficiency and simplicity of the design process. Fuzzy logic renders superfluous the requirement for precise mathematical models when overseeing production facilities or determining the optimal course of action. This makes the development of system controller programmes faster and easier. This seems to imply, intuitively, that such input variables are approximated by the brain, for instance, in the assessment of condition verification levels within fuzzy logic. It consists of a fuzzifier, an inference engine, a data base, a rule base, and a defuzzifier. Several types of member functions are employed, including trigonometric functions, trapezoidal functions, generalised bell-shaped functions, Gaussian curves, polynomial curves, and S-shaped functions. Figure 3 displays the triangle membership function in this study. The triangular curve depends on three parameters, a, b, and c, and is expressed as:

(4) fx;a,b,c=0       x≤a                            x−bb−a       a≤x≤b                            c−xc−b       b≤x≤c                            0       x≥c      

**Figure 4:**
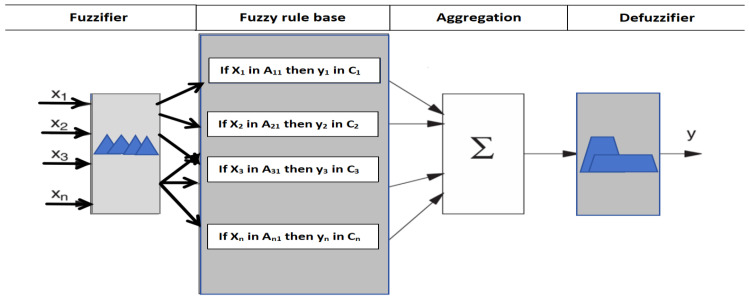
A procedure of fuzzy reasoning using a Mamdani fuzzy system with n inputs and a knowledge base with “if–then” rules.

The input fuzzy sets are described by their triangular membership functions, which define three fuzzy sets within the input domains. Each input signal was classified into three fuzzy subsets: low (L), medium (M) and high (H), as shown in Figure 3a. The multi-grey fuzzy indices present multiple triangular-shaped fuzzy sets within the output domain. The output signal is divided into nine fuzzy subsets: Tiny (T), Very Small (VS), Small (S), Small–Medium (SM), Medium (M), Medium–Large (ML), Large (L), Very Large (VL) and High (H), as illustrated in Figure 3b. The Mamdani implication method allows easier interpretation of the relationship of inputs and responses to the fuzzy rule base, which consists of a set of IF–THEN production rules. There are two distinct steps involved in interpreting an if–then rule. The antecedent, or premise, of a rule is the if part, “x is A”, while the consequent, or conclusion, of a rule is the then part, “y is B”. The Mamdani implication method was adopted for the fuzzy inference reasoning in this study. As shown in Figure 4, in fuzzy logic, when processing antecedents, the antecedents must first be fuzzified, that is, explicit input values are converted into membership degree values of fuzzy sets via membership functions. Fuzzy operations include intersections, unions, complements, and other manipulations of fuzzy sets, such as MIN-MAX operations. The results of an antecedent that is fuzzified are then applied to the consequent through fuzzy inference rules. Finally, the outcome of fuzzy reasoning is defuzzified by evaluating the membership function, which yields a readable conclusion. A typical fuzzy rule, such as ‘IF (x is A_i_ and y is B_i_) THEN (z is C_i_)’, which is used in Mamdani inference systems and is shown in Figure 4, can be implemented through a fuzzy relation R_i_, which is defined by the membership function μ_Ri_, as shown in Equation (1). For the R_i_ rule,

(5) $$R_{i}:\;\mathrm{If}\;x_{1}\;\mathrm{is}\;A_{i1},\;x_{2}\;\mathrm{is}\;A_{i2},\ldots ,\;\mathrm{and}\;x_{s}\;\mathrm{is}\;A_{is},\;\mathrm{then}\;y_{i}\;\mathrm{is}\;C_{i},\;i\;=\;1,\;2,\;\ldots ,\;M,$$

where M is the total number of fuzzy rules, x_j_ (j = 1, 2,…, s) are the input variables, y_i_ are the output variables, and $A_{ij}$ and $C_{i}$ are fuzzy sets characterised by membership functions $\mu_{A_{ij}}\left(x_{j}\right)$ and $\mu_{C_{i}}\left(y_{i}\right)$, respectively. A set of disjunctive rules is based on the Mamdani inference method. In this method, the conjunction “and” of the antecedent takes the minimum value of the T-norm (Min). The grey correlation degree is evaluated in this paper. This is a measure of the grey relational coefficient of the responses for each system in the fuzzy logic system. It obtains weights through fuzzy rules in the inference engine based on the given input information. By way of example, the output of the ith rule in Equation (6) can be found by performing an inference operation.

(6) $$w_{i}=\underset{i}{\min}\left(\mu_{A_{i1}}\left(x_{1}\right),\mu_{A_{i2}}\left(x_{2}\right),\ldots ,\mu_{A_{\mathrm{is}}}\left(x_{s}\right)\right)$$

where w_i_ is the strength with which the ith rule fires. Also, the results of reasoning for various rules are aggregated by applying the s-norm maximum (Max) function. Accordingly, the aggregated output for the M rules is

(7) $$\mu_{C_{i}}\left({\bar{y}}_{i}\right)=\mathrm{Max}\left[\underset{i}{\min}\left(\mu_{A_{i1}}\left(x_{1}\right),\mu_{A_{i2}}\left(x_{2}\right),\ldots ,\mu_{A_{\mathrm{is}}}\left(x_{s}\right)\right)\;i=1,2,\ldots ,M.\right]$$

The grey relational grade ($\gamma_{i}$) can therefore be calculated as follows:

(8) $$\gamma_{i}=\sum_{k=1}^{n}\mu_{C_{i}}\xi_{i}\left({\bar{y}}_{i}\left(k\right),{\bar{y}}_{j}\left(k\right)\right)\;i=1,2,\ldots ,n.$$

**Figure 5:**
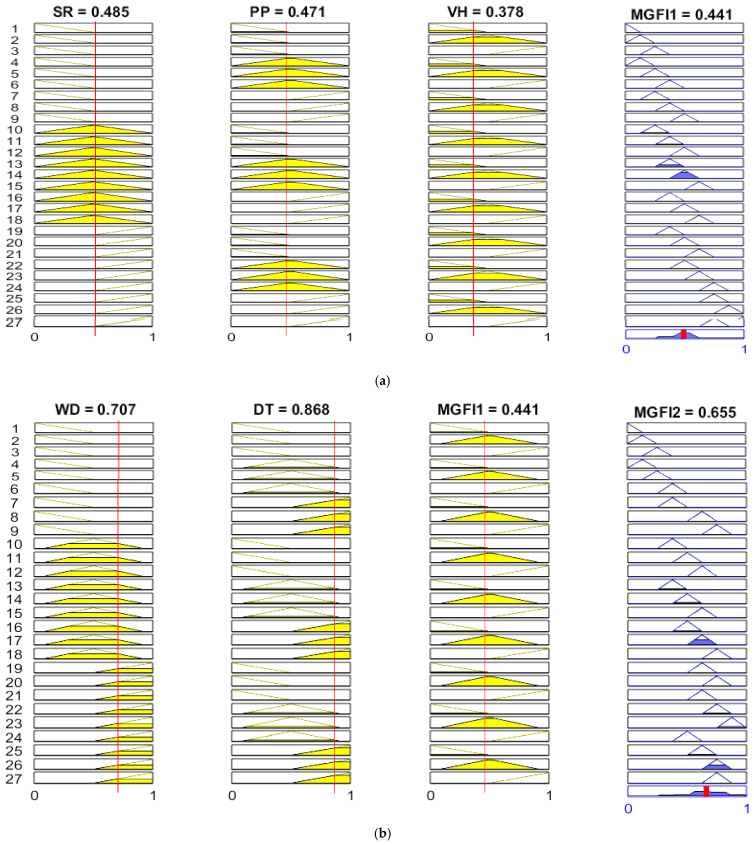
The multi-response behaviour exhibited in fuzzy inference mechanisms through establishing “if–then” rule bases, including input variables and their grey relational coefficients, among which (**a**) MGFI1 contains SR, PP, and VH, and (**b**) MGFI2 contains WD, DT, and MGFI1.

The five input variables that are used in this study are surface roughness (SR), porosity percentage (PP), Vickers hardness (VH), wear depth (WD) and deposit thickness (DT). The rule database of this study consists of five input variables, each of which is divided into three fuzzy sets, and one output variable containing nine fuzzy sets. The fuzzy inference engine is made using an “IF–THEN” rule database and works through Equations (6)–(8). A total of 54 rules are contained in the rule database, which is made up of two fuzzy logic systems, each comprising 27 rules. The numbers that go into this fuzzy system are the grey relational coefficients of SR, PP, VR, WD, and DT values. These five input signals are grouped into two sets: MFGI1 (SR, PP and VR) and MFGI2 (WD, DT and MFGI1). The graphical program and results for Test 1, created using MATLAB R2024b, are shown in Figure 5. The rows of the figure represent 27 rules, and the columns represent three input variables. The rightmost column in Figure 5 shows the output signals and reveals nine subsets: T, VS, S, SM, M, ML, L, VL and H. Multiple rules can be triggered to generate fuzzy linguistic values for the output response by applying rule-based systems and Mamdani inference engines. This study constructed two knowledge bases using fifty-four rules, which were then employed to compute five attributes through fuzzy logic reasoning, as shown in Figure 1. By integrating logical rules with the Mamdani inference procedure, fuzzy linguistic values and their membership degrees for the output response were obtained. First, a knowledge base is constructed using 27 rules that employ fuzzy logic reasoning to calculate integrated indicator values for three attributes. These values are then combined with the latter two attributes to create another knowledge base using an additional 27 rules. Integrating logical rules with the Mamdani inference procedure yields fuzzy linguistic values and their membership degrees for the output response. For example, Test 1 in Table 2 shows that the grey relational coefficients for surface roughness (SR), porosity (PP) and hardness (VH) are 0.485, 0.471 and 0.378, respectively. Figure 5a shows that, during the fuzzy inference system, the grey relational coefficients for surface roughness (SR), porosity percentage (PP) and Vickers hardness (VH) within the 27-rule knowledge base were analysed. This resulted in 10–18 rules for SR; 4–6, 13–15 and 22–24 for PP; and 1–2, 4–5, 7–8, 10–11, 13–14, 16–17, 19–20, 22–23 and 25–26 for VH. Its value is 0.441. These rules are triggered simultaneously through the fuzzy rule-based inference process. There are four rules that activate when all three MGFI1 attributes are triggered simultaneously. These rules are as follows:

Rule 10: If (SR is “M”, PP s is “M”, VH is “M”) Then (MGFI1 is “SM”)
Rule 11: If (SR is “M”, PP s is “M”, VH is “M”) Then (MGFI1 is “SM”)
Rule 7: If (SR is “M”, PP s is “M”, VH is “M”) Then (MGFI1 is “SM”)
Rule 8: If (SR is “M”, PP s is “M”, VH is “M”) Then (MGFI1 is “ML”)

The aforementioned MGFI1 values were subsequently combined with the latter two attributes, wear depth (WD) and deposit thickness (DT). This was done to establish another knowledge base. This knowledge base used an additional 27 rules. The grey relational coefficients for WD, DT and MGFI1 are 0.707, 0.868 and 0.441, respectively. The inference analysis of grey relational coefficients for WD, DT, and MGFI1 within the 27-rule knowledge base in the fuzzy system is shown in Figure 5b. Individual attribute analysis yielded the following rules: the rules for WD are 19–27; for DT, they are 4–9, 13–18, and 22–27; and for MGFI1, they are 1–2, 4–5, 7–8, 10–11, 13–14, 16–17, 19–20, 22–23, and 25–26. Its value is 0.655. These rules are triggered simultaneously through the fuzzy knowledge base. The following describes scenarios in which the three attributes and the eight aforementioned rules (13–14,16–17,22–23, and 25–26) are triggered simultaneously:

Rule 13: If (WD is “M”, DT s is “H”, MGFI1 is “M”) Then (MGFI2 is “SM”)
Rule 14: If (WD is “M”, DT s is “H”, MGFI1 is “M”) Then (MGFI2 is “M”)
Rule 16: If (WD is “M”, DT s is “H”, MGFI1 is “M”) Then (MGFI2 is “M”)
Rule 17: If (WD is “M”, DT s is “H”, MGFI1 is “M”) Then (MGFI2 is “ML”)
Rule 22: If (WD is “H”, DT s is “H”, MGFI1 is “M”) Then (MGFI2 is “VL”)
Rule 23: If (WD is “H”, DT s is “H”, MGFI1 is “M”) Then (MGFI2 is “H”)
Rule 25: If (WD is “H”, DT s is “H”, MGFI1 is “M”) Then (MGFI2 is “L”)
Rule 26: If (WD is “H”, DT s is “H”, MGFI1 is “M”) Then (MGFI2 is “VL”)

### 3.4. The Grey Relational Analysis

Grey relational analysis is one of the core analytical methods of grey system theory, which was introduced by Chinese scholar, Professor Deng Julong in 1982 [45]. It addresses the problem of quantifying the correlation between multiple factors within uncertain systems where some information is known, and some is unknown. Grey relational systems constitute a methodology for addressing uncertainty, capable of generating precise predictions with minimal data requirements. They offer several advantages, including reduced parameter design datasets for comparison and efficient attainment of optimal solutions. Consequently, grey relational analysis grounded in grey system theory is employed to resolve intricate interrelationships among multiple responses. The method assesses the strength of relationships between factors by comparing the geometric similarity of “reference sequences” (target variables) with “comparison sequences” (influencing factors). The closer the curves are aligned, the higher the correlation is; conversely, the weaker the relationship is, the lower the alignment. Grey relational analysis is employed to measure the correlation between reference sequences and test sequences. To calculate the absolute difference sequence, we first determine the absolute difference between the comparison and reference sequences at each point in the sample.

(9) $${∆}_{oi}\left(k\right)=\left|X_{o}\left(k\right)-X_{i}\left(k\right)\right|\;i=1,2,,\ldots ,m;k=1,2,\ldots ,p$$

where *X_0_(k)* is the reference sequence, while *Xᵢ(k)* is the comparison sequence. To determine the extremes, the minimum value Δmin (the lesser of the two differences) and the maximum value Δmax (the greater of the two differences) among all absolute differences are identified.

$$∆min=\underset{\forall k}{\min}\;\underset{\forall i}{\min}{∆}_{i}\left(k\right)\;∆max=\underset{\forall k}{\max}\;\underset{\forall i}{\max}{∆}_{i}\left(k\right)$$

ς is a user-selectable coefficient, $\varsigma \in \left[0,1\right]$; the smallest value is picked out from all the differences that are shown in the groups. The groups Δ_i_(k) are shown by i, and the elements are shown by k. As mentioned above, the reference sequence is the best quality characteristic. If the ${∆}_{oi}\left(k\right)$ is closer to the Δmin, then $\xi_{i}\left(x_{i}\left(k\right),x_{j}\left(k\right)\right)$ is approximately one. The correlation coefficient may be calculated using the following formula.

(10) $$\xi_{i}\left(x_{i}\left(k\right),x_{j}\left(k\right)\right)=\frac{\Delta \min+\varsigma \Delta \max}{\Delta_{\mathrm{oi}}\left(k\right)+\varsigma \Delta \max}$$

The local association is reflected by the relational coefficient, while the overall degree of association is synthesised by the relational degree. The weighted average method is a commonly used approach. Its weight is calculated through inference by the fuzzy logic system. The calculation of grey relational degree ($\gamma_{i}$) is as follows:

(11) $$\gamma_{i}=\sum_{k=1}^{n}w_{k}\;\times \;\xi_{i}\left(x_{i}\left(k\right),x_{j}\left(k\right)\right),{\;\;\;\;\;\;w}_{1}+w_{2}+\cdots +w_{n}=1\;$$

The composite result $\gamma_{i}$ for the ith object is the weighted sum of observations from n samples. In other words, it is the sum of the observations from the *n* samples, each observation being weighted by its respective weight w_k_, where *w_k_* is the weight of the kth sample. There are twenty-seven rules in the fuzzy rule base of the fuzzy inference system, which uses triangular membership functions and five input variables. The gravity method was used for defuzzification in this work. The defuzzification value is computed using Equation (12), which is referred to as the Multi-Grey-Fuzzy Index (MFGI). The formula for finding the centroid of the outputs of the aggregated rule, MFGI_i_, i = 1,2 is given by:

(12) $$MGFI_{i}=\frac{\sum_{k=1}^{n}\mu_{C_{i}}\xi_{i}\left({\bar{y}}_{1}\left(k\right),{\bar{y}}_{j}\left(k\right)\right)}{\sum_{i=1}^{n}\mu_{C_{i}}\left({\bar{y}}_{i}\right)}$$

### 3.5. Estimation of SNRs and ANOVA for Grey-Based Fuzzy Systems

This study extends Taguchi methods by introducing the signal-to-noise ratio (SNR) concept from the field of audio engineering into multivariate experiments. The SNR formula is derived through calculating grey relational grade, wherein grey relational grades employ the fuzzy rule base of the inference engine to compute the weights of each grey relational coefficient, thereby synthesising an overall comprehensive grade. The calculation of the SNR is shown in Table 4. To obtain optimal experimental results, users may select the largest numerical value. In this paper, the SNR evaluation that weights the grey relational coefficient of the responses for each grey system in the fuzzy logic is expressed as follows:

(13) $${SNR}_{i}=-10\log\left(\sum_{i=1}^{n}{\left(\frac{1}{MGFI2}\right)}^{2}\right),\;\;\;\;\;\;i=1,2,\ldots ,n;$$

The SNR of MGFI2 is optimised by integrating properties as the multi-grey fuzzy index increases. In other words, the higher the SNR, the more effectively the sprayed system is performing its intended function, regardless of noise factors. In other words, the system is more robust against noise. The SNR of MGFI2 is calculated using Equation (13). As the maximum average value is reached, the parameter levels are optimised.

(14) $${SNR}_{B1}=\frac{1}{6}\left({SNR}_{1,2,3}+{SNR}_{10,11,12}\right)\;{SNR}_{B2}=\frac{1}{6}\left({SNR}_{4,5,6}+{SNR}_{13,14,15}\right)\;{SNR}_{B3}=\frac{1}{6}\left({SNR}_{7,8,9}+{SNR}_{16,17,18}\right)$$

## 4. Experimental Results and Discussion

### 4.1. Surface Performance of Coatings

The results of each test for the plasma spray coatings and the calculations using the SNR are shown in Table 3 and Table 4. The surface roughness ranged in value between 6.96 and 10.01 μm, which averaged out at 8.21 μm. Test 8 showed the minimum value of 6.96 μm, while Test 5 showed the maximum value of 10.01 μm. The variation in surface roughness was minimal, reflecting a notably flatter surface. In other words, the surface roughness is satisfactory. The percentage change in porosity varies significantly, ranging from 6.72% to 20.29%, with an average value of 12.35%. Test 11 exhibited the lowest porosity, while Test 10 showed the highest value. This result indicates the presence of a certain proportion of pores on the coating surface, which is primarily attributed to the residual rate of unmelted/melted particles during plasma spraying. Additionally, the wear depth values ranged from 43.70 μm to 153.66 μm, with an average of 65.78 μm. The 13th test yielded the minimum value, while the 7th test produced the maximum value. This indicates significant variation in the wear depth of the coating. The softer coating structure wears more quickly than the harder zirconia structure of the coating, which wears more slowly. Therefore, this hard structure results in an extremely low wear volume for the coating. The coating hardness ranges from 890 Hv to 1484 Hv, with an average value of 1201 Hv. Test 6 exhibited the lowest hardness value, while Test 14 produced the highest hardness value. This demonstrates that the coating exhibits significantly higher hardness, exceeding that of the substrate by approximately 4 to 5 times. The coating thickness varied between 46.33 μm and 211.83 μm, with an average of 76.23 μm. Test 7 exhibited the lowest thickness, while Test 13 showed the highest. This indicates significant variation in coating thickness, likely related to heat input. An excessively thick coating can cause stress concentration and lead to peeling, thus requiring an appropriate thickness. As detailed in Table 3, the five highest quality values, as determined by normalised calculations, indicate that they belong to different experimental groups due to differences in individual characteristics. Only a single quality can be optimised, while multiple qualities cannot be optimised. Therefore, the above-mentioned five properties of the coating cannot be applied in industry. Further processing is required.

### 4.2. Surface Morphology of Coatings

**Figure 6:**
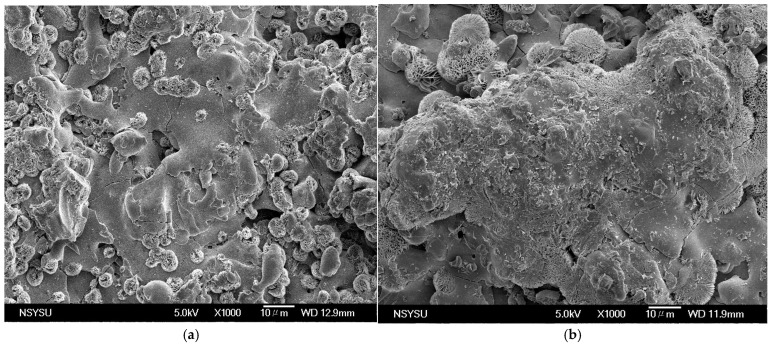

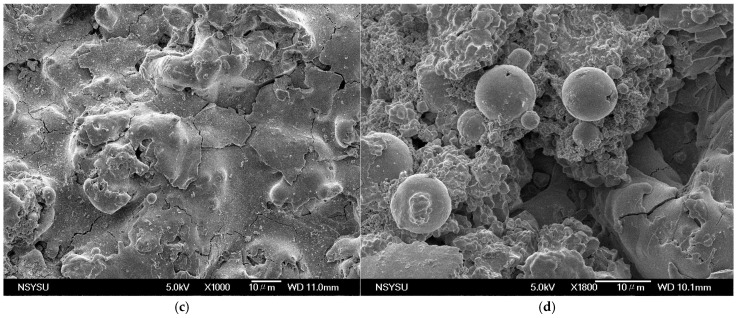
Typical microstructures of sprayed zirconia coatings after SEM inspection: (**a**) Trial 3, (**b**) Trial 5, (**c**) Trial 8, and (**d**) Trial 15.

Figure 6 illustrates the typical microscopic structure of a zirconia coating after spraying as observed by scanning electron microscopy. It reveals the distribution of aggregates formed by multiple flat splats stacked within the coating, along with the interlaced lamellar features that result from incomplete spreading and multi-layer stacking of the splats. Figure 6a shows that the majority of the ZrO_2_ in the melt zone has melted completely, which has resulted in a smoother surface on the splat aggregate. The texture is more consistent, and there are no distinct particles adhering to the surface. Only the inherent irregularities and fractured patterns of the splat itself are visible. Figure 6b shows that the molten region largely exhibits a porous texture, which is formed by loosely agglomerated fine particles. This textured surface is created by non-melted spherical particles of ZrO_2_. The lower-right area has large pores and a few completely melted droplets without significant cracks; however, the overall structure is more discrete and porous. Figure 6c shows a splat structure dominated by molten zirconia in the central region, with minute particles adhering to its surface. The particles exhibit a needle-like texture. This structure is actually an aggregate of needle-like crystals from the metastable tetragonal phase of zirconia (t-ZrO_2_) [24]. This phenomenon occurs when small particles partially melt and adhere to the splat surface during the rapid cooling of molten zirconia at high temperatures. This rapid cooling process induces nucleation and growth, resulting in the formation of these needle-like crystals. Several larger pores are distributed around the splat, and interlayer pores can be observed. As shown in Figure 6d, the large splat aggregates in the coating are approximately 20–50 μm in size. These aggregates form stacked layers of multiple splats, which are surrounded by acicular eutectic-like structures. This phenomenon primarily results from a lower cooling rate. The interfaces between the splats are clearly visible and are accompanied by numerous interlayer pores (0.5–2 μm). These voids primarily result from incomplete filling of the interfacial gaps during droplet spreading, as well as from the spaces left behind by rapid solidification. A number of pores are distributed around larger splats, and microcracks can be detected. In summary, most melted particles in the fusion zone exhibit a flattened or lamellar shape, formed when molten particles rapidly spread and cool upon impact with the substrate. In some areas, spherical or near-spherical particles are visible. These particles may represent powder that did not fully melt during the spraying process, and their presence can affect the coating’s density and performance. Additionally, cracks are primarily distributed at the lamellar interfaces and particle boundaries. The morphology and distribution of these cracks significantly influence the coating’s thermal shock resistance.

### 4.3. Cross-Section Microstructure

**Figure 7:**
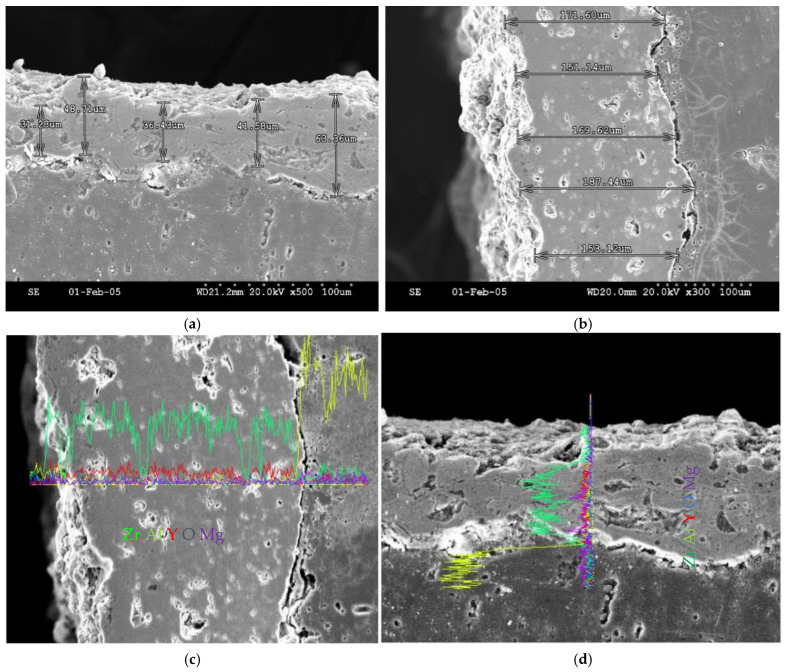
The cross-sectional images of the microstructure of the unetched zirconia composite coating produced by the plasma spraying process, including porosity, thickness, EDS and unmelted zirconia particles, from (**a**) Trial 2, (**b**) Trial 16, (**c**) Trial 9, and (**d**) Trial 15.

The cross-sectional image in Figure 7 shows the ZrO_2_ composite coating produced by the plasma spray process. There are still unmelted ZrO_2_ particles on the surface of the coating. The melted zone contains pores of various sizes, which account for around 5–10% of the area. There is a crack at the interface between the melted zone and the substrate containing unmelted particles. Impact-induced flaking is evident in certain areas of the substrate. This interface region appears to exhibit mechanical bonding. Figure 7a shows molten droplets impacting the substrate at different angles during spraying. There are significant fluctuations in thickness (30.26 μm to 65.56 μm) exhibited by different regions. The interface has an uneven morphology where the coating bonds to the substrate through mechanical adhesion. Upon impact, minor portions of the molten droplets embed into rough surface areas, resulting in physical adhesion. No significant through-cracks are visible in the observed regions. The coating has a layered, stacked structure due to the sequential deposition of flattened droplets (splats). This results in fine internal pores that are visible. Moreover, the coating surface evinces a texture marked by unevenness and the presence of granular protuberances. This is principally attributable to droplets that have not been fully flattened or to partially melted powder particles. As shown in Figure 7b, the coating thickness varies significantly, with a range of approximately 46 μm. The overall coating thickness is found to be between 151.34 μm and 197.44 μm. This reflects the randomly stacked droplets of molten material during plasma spraying, which is a process that involves the application of high-temperature, high-pressure jets of material to a surface to harden it. It results from factors such as the movement trajectory of the spray gun and the uneven powder feed rate. Visible internal porosity includes layered voids between splat layers and gas pores trapped during droplet solidification, with a relatively dispersed distribution. Microcracks exist at the coating–substrate interface, which exhibits an irregular serrated profile. Localised microcracks occur in specific areas of the substrate interface. The bond between the coating and substrate is mechanical, with no metallurgical bonding features at the interface. The concave–convex morphology of the bonding interface is due to the adhesion effect of droplets, but the bond strength is weakened by microcracks at the interface. The surface of the coating (illustrated on the left) is uneven, displaying amorphous accumulations of droplet particles that have not been. Figure 7c shows that the coating on the left exhibits the typical layered porous structure of plasma spraying, where dispersed pores are formed by the interstices between stacked droplets. The uneven interface between the coating and substrate (right region) is typical of the mechanical adhesion nature of plasma spraying. A distinct crack is visible on the right-hand side of the interface, which is primarily an interfacial cracking defect caused by thermal cycling stresses during spraying deposition. As indicated by EDS analysis, the green curve for Zr elements shows significantly higher concentrations in the left coating region, while the yellow curve shows higher Al concentrations in the substrate region on the right. However, the concentrations of other elements are relatively low. As shown in Figure 7d, a partially melted, highly agglomerated structure that is notably rough is exhibited by the surface of the ZrO_2_ coating. Dispersed micro-voids are distributed within the coating. These are formed by stacked gaps between flattened splats and residual pores from solidification. These voids enable the thermal barrier coating to reduce thermal conductivity effectively. The surface where the coating meets the substrate is uneven and shows signs of adhesion but not of a chemical change. The coating interior contains only dispersed micro-voids. There is no extensive cracking. Despite the presence of minute gaps at the interface, no through-cracking is evident. The green curve representing Zr in Figure 7c is similar to the EDS elemental analysis. It shows a higher concentration in the coating. The yellow curve for the Al substrate exhibits a significantly higher content. The localised variations in composition within the coated region are caused by microstructural inhomogeneities in partially melted ZrO_2_, which are reflected by the Zr element in the EDS analysis.

### 4.4. Wear Behaviour of Coatings

**Figure 8:**
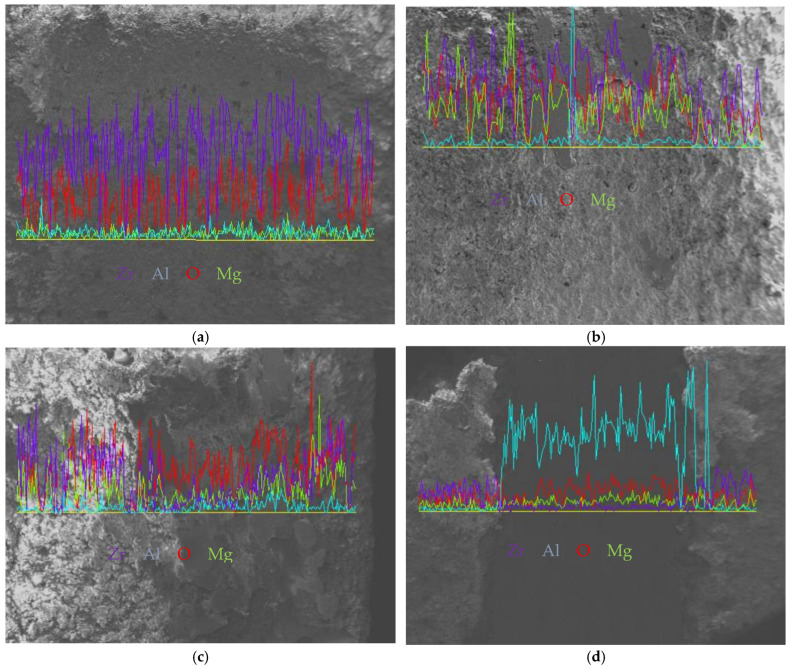
SEM images showing wear behaviour and EDS maps of elemental distribution for plasma-sprayed coatings subjected to wear testing: (**a**) Trial 2, (**b**) Trial15, (**c**) Trial 6, and (**d**) Trial 18.

Figure 8a illustrates the layered porous ZrO_2_ coating subjected to abrasion. Under wear action, the surface splat undergoes microfracturing and spalling. The pores become filled with abrasive particles. This creates a coarse yet uniform worn surface. The texture exhibits consistent undulations across the entire observation area, indicating that the coating wears uniformly without any localised excessive wear or spalling. The coloured curves in the EDS correspond to labelled elements. Zr is purple, Al is blue, O is red, and Mg is yellow. These reflect compositional fluctuations on the worn surface. The EDS image also shows these changes in the wear surface. The different colours in the image show changes in the elements present. The purple/red curves typically correspond to Zr and O elements. These are the primary metallic elements in ZrO_2_. The curves show dense fluctuations. These result from wear. This exposes different splat layers and porous regions within the coating. This causes localised variations in Zr content. This indicates that the material exposed after wear remains predominantly the ZrO_2_ coating. The green curve in the figure represents the presence of aluminium, which is present in extremely low and stable amounts. This suggests that the wear depth is likely to remain confined within the coating itself and has not yet penetrated the substrate. Figure 8b shows that the surface of the coating has a dense and irregular, plough-like texture. The pattern is caused by the abrasive particles moving across the coating surface, which leads to micro-cutting and ploughing actions on the surface material. The layered, porous and brittle structure of ZrO_2_ also contributes to microfracturing and spalling of the splat, which is a typical example of abrasive wear. No signs of localised excessive wear or large-scale spalling were observed. EDS analysis of the worn surface revealed that the dominant elements were Zr and O, with no significant exposure of substrate elements. This indicates that wear penetration remains confined within the coating and does not extend to the substrate. Furthermore, the absence of extensive coating spalling suggests good adhesion between the coating and substrate. The predominant form of wear is abrasive, accompanied by brittle spalling. As shown in Figure 8c, the worn surface of the coating is uneven and rough, with some areas raised and others recessed. This is primarily due to the abrasive sliding, which causes micro-cutting and ploughing effects on the surface layer of the layered, porous, brittle ZrO_2_ structure. Under wear stress, these effects propagate, leading to localised material spalling. These are typical characteristics of abrasive wear and brittle spalling. As can be seen in the image, localised areas, such as the recessed wear on the right side, exhibit more pronounced wear. This may be related to structural inhomogeneities within the coating itself, such as localised high porosity or weak splat bonding. The coloured curves in the EDS correspond to labelled elements (Zr = purple, Al = blue, O = red, Mg = yellow), reflecting compositional fluctuations on the worn surface. The Zr (purple) and O (red) curves exhibit highly synchronised fluctuations. These are at peak concentrations. This confirms ZrO_2_ coating as the primary exposed material post-wear. The dense fluctuations in the curves originate from the layered structure within the coating. This is exposed during wear. It leads to local variations in Zr/O content. These variations are caused by different splat layers and pore regions. The Al (blue) curve displays a notable peak, with reduced content observed in other regions. This may be due to the exposure of Al_2_O_3_-doped phases added to the coating during wear, or to the retention of abrasives (e.g., Al_2_O_3_) on the coating surface. The Mg (yellow) curve shows a very low and steady content, which indicates that there are trace impurity elements in the coating or substrate that do not have a big effect on wear performance. Figure 8d shows an uneven surface texture with larger roughness on both edges and a concave pattern in the centre. The central region exhibits severe abrasive wear with pronounced plough marks and significant spalling. Compared to the peaks mentioned in the EDS images, the Al peak clusters exhibit distinct morphological anomalies characterised by elevated protrusions. This is primarily due to the non-uniform, layered, porous and brittle structure of ZrO_2_, which propagates under abrasive stress. This leads to material spalling in this region, ultimately forming this distinct wear surface. This indicates poor uniformity in wear behaviour, with more pronounced wear occurring in localised areas. The coloured curves in the EDS correspond to labelled elements, reflecting compositional fluctuations across the worn surface. The Zr (purple) and O (red) curves show low-level synchronised fluctuations with minimal content, indicating that the exposed substrate remains predominantly Al-based after wear. The dense undulations in the curves originate from the removal of the inner coating layer during wear, leading to localised variations in Zr/O content. The Al (blue) curve exhibits a distinct cluster of peaks in the middle, with lower concentrations elsewhere. This may indicate that the ZrO_2_ phase within the coating was not apparent during wear or that abrasive particles are not visible on the coating surface. Mg (yellow) shows extremely low concentrations. It also shows smooth fluctuations. This indicates that it is a trace impurity element within the coating or substrate. It exerts no significant influence on wear performance.

### 4.5. Normalisation

The aforementioned report indicates that the five properties, due to their differing natures, which have been normalised, fall between 0 and 1. A value of 0 represents the worst value, while 1 represents the best value. The integrated quality characteristics in the ZrO_2_ coatings for the parameter design are represented in terms of surface roughness, porosity, hardness and wear volume. The experimental results of plasma spray tests are listed in Table 3. The performance of each experimental setting is evaluated not only by grey relational analysis but also by the SNR of its integrated properties. In the grey relational analysis, initially, each experimental datum is normalised between 0 and 1. The grey relational coefficient that shows the relationship between the desired and experimental datum is calculated as shown in Table 4. Accordingly, the SNR is calculated by applying fuzzy logic to weight the grey relational coefficients of each response variable, and then performing the relevant conversion based on grey relational grades. Notably, all attributes are normalised to fall between 0 and 1, while all properties maintain consistent directionality. Based on the above results, the closer the grey relational rank is to the desired value of 1, the higher the SNR becomes, thereby leading to optimal characteristics.

### 4.6. Estimation of the Impact of SNR Values on Coatings

The conduction of each test in this experiment involved two duplicate measurements for the purpose of the evaluation of experimental performance. There were five characteristic tests measuring surface roughness, porosity percentage, hardness, wear depth and deposit layer thickness. The overall composite rating was derived through grey relational grading calculations. The weights of each grey relational coefficient were computed using the fuzzy rule database of the inference engine, thereby synthesising the overall integrated index and SNR formula calculation. The results of the overall integrated index and SNR are detailed in the right-hand column of Table 4. The ideal quality characteristics for integrated performance are expected to be achieved by the optimal coating application process. These include the highest hardness level, the desired coated thickness values, and the lowest surface roughness, porosity percentage, and wear depth. By analysing the SNR and average values (listed in Table 5), the maximum SNR value for the zirconia coating can be identified, which represents the optimum setting for the control parameters. These are the spraying layers (A) at level 1, the accelerating voltage (B) at level 3, the arc current (C) at level 2, the travel speed (D) at level 1, the stand-off distance (E) at level 1, the powder feeder rate (F) at level 3, the carrier gas (G) at level 1 and the primary gas (H) at level 2. In addition, the quality characteristics of integrated properties are significantly affected by control factors, which are explored by the analysis of variance. The results of the variance analysis for multi-quality characteristics of zirconia coatings are shown in Table 6, which also provides further information on the analysis. The following percentages of the total variance in integrated performance were accounted for by the influence parameters, respectively, along with their corresponding significance levels: accelerated voltage (26.70%), spray distance (21.05%), powder feed rate (10.95%), spray distance (10.52%), and travel speed (8.74%). The remaining parameters exhibited smaller effects. Together, the significant parameters accounted for approximately 77.96% of the total variance in integrated performance.

### 4.7. Graphical Visualisation of Fuzzy Inference Based on Optimised Grey Relational Coefficients

**Figure 9:**
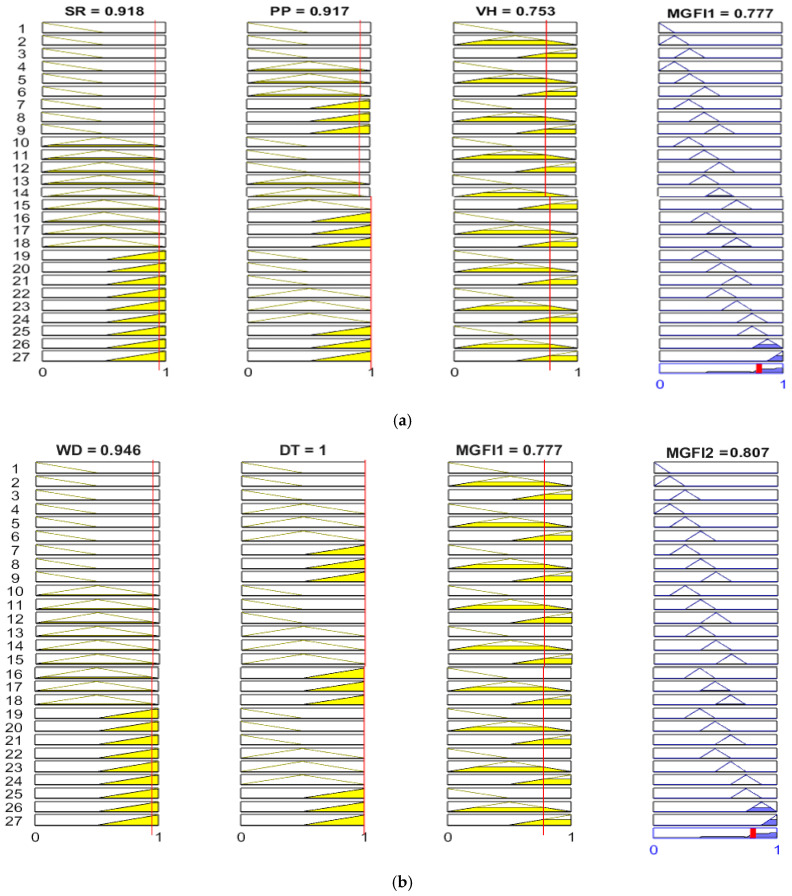
The rule-based array used for the fuzzy logic system with two rule-base inferences: (**a**) MGFI1, (**b**) MGFI2.

As can be seen from Equation (4), the rules listed in Figure 3 are preferred. To find an approximate solution for multi-response attributes, a graphical inference interface is used to perform computations with five sets of different grey relational values as inputs. As shown in Figure 9, the Mamdani inference method involves two sets of analysis for the “if–then” rule database within the fuzzy control system, based on the simplified rule base. The rule bases for the first set are analysed as follows: SR triggers nine rules (e.g., 19–27), PP triggers eight rules (e.g., 7–9, 16–18, 25–27) and VH triggers eighteen rules (e.g., 2–3, 5–6, 8–9, 11–12, 14–15, 17–18, 20–21, 23–24, 26–27). Based on the Mamdani inference method illustrated in Figure 9, the generation of only eight rules by MGFI1 through fuzzy calculation (e.g., 14–15, 17–18, 23–24, and 26–27) is evident, with distribution in the deeply blue area on the right side of Figure 9. The final calculated MFGI1 value of 0.777 is displayed in the top right-hand corner of Figure 9a. In addition, the analysis matrix for group 2 rule databases shows 9 rules triggered by WD (e.g., 19–27), 8 rules triggered by DT (e.g., 7–9, 16–18, 25–27), and 18 rules triggered by MGFI1 (e.g., 2–3, 5–6, 8–9, 11–12, 14–15, 17–18, 20–21, 23–24, and 26–27). Based on the Mamdani inference method illustrated in Figure 9, the MGFI2 system generates only four rules (e.g., 17–18, 26–27) through fuzzy calculation, which are distributed in the deep blue area on the right side of Figure 9b. The MGFI2 value calculated via fuzzy computation is 0.807. This was obtained through the fuzzy inference mechanism illustrated in Figure 9. Upon undertaking a comparison between optimal settings and the remaining 18 test data sets in Table 4, it can be verified that the optimised MGFI2 for multiple quality characteristics is more proximate to 1, as illustrated in Table 4. The higher MGFI2 values observed in this study suggest superior multi-response characteristics. Specifically, higher MGFI2 values signify superior performance in terms of SR, PP, VH, WD and DT. Furthermore, the greater the SNR of the inference values from the grey-based fuzzy, the better the multi-response performance is. Therefore, it can be concluded that the grey-based fuzzy effectively predicts multi-response characteristics.

### 4.8. Effect of Relational Coefficients on the Multi-Properties

**Figure 10:**
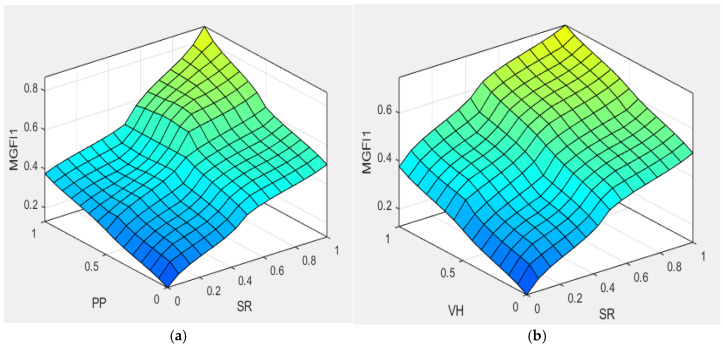

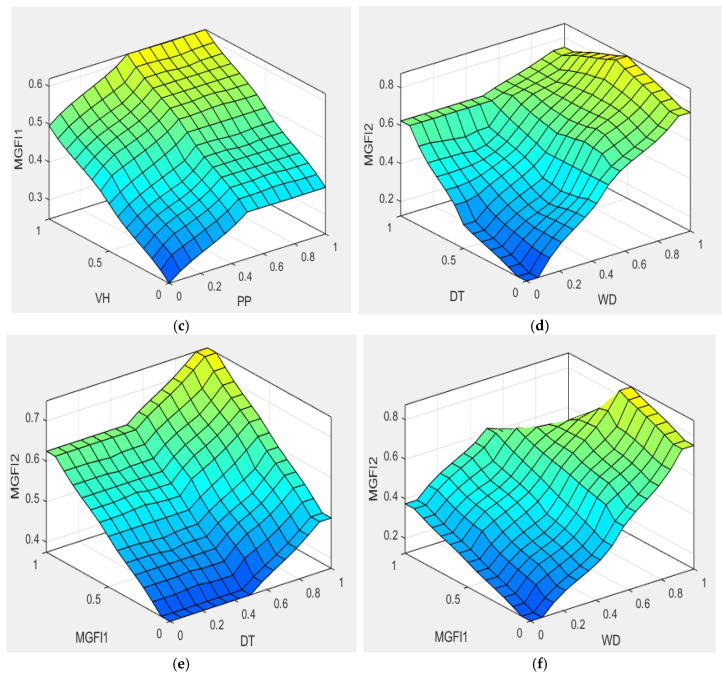
The impact of different factorial settings on multiple properties of spray-coated zirconia coatings. (**a**) SR and PP, (**b**) PP and VH, (**c**) SR and VH for MGFI1, and (**d**) WD and DT, (**e**) WD and MGFI1, (**f**) DT and MGFI1 for MGFI2.

The illustration in Figure 10 shows how different parameters in fuzzy inference rule bases affect the multi-properties of ceramic coatings. The rule bases are simplified by examining the effects of parameter variations on MGFI1 and MGFI2 in sequence. The figure shows that, within the experimental space, the multi-properties of ceramic coatings exhibit curved surfaces that reflect the differing influence of various factors. The influence of parameters in the process of fuzzy inference for obtaining multi-properties of ceramic coatings can be illustrated from a two-dimensional surface diagram, which is a useful tool for visualising such information. The influence of five parameters (properties) in ceramic coating spraying on multiple performance attributes is illustrated by this figure, where normalised values between 0 and 1 are visualised. The findings elucidate the manner in which each parameter influences the overall performance of the coating. As shown in Figure 10a, a surface on MGFI1 is yielded by changes in SR and PP. The blue region has the lowest MGFI1 values, while the yellow–blue region has the highest. For SR values in the range of 0–1, an increase in MGFI1 with increasing PP is seen. Similarly, for PP values in the range of 0–1, MGFI1 increases with increasing PP. This is within the experimental range. In other words, it was found that better multi-properties are yielded by larger values of both SR and PP. The surface generated by SR and VH on MGFI1, as shown in Figure 10b, approximates that in Figure 10a. Between SR values of 0 and 1, MGFI1 increases with growing VH, while between VH values of 0 and 1, MGFI1 rises with increasing SR within the experimental domain. Figure 10c shows the surface generated by PP and VH on MGFI1. The MGFI1 value reaches its maximum when PP increases to 0.5–1. When PP is in the range of 0–1, the MGFI1 value increases with increasing VH. This indicates that VH has a greater influence than PP in multi-properties. Figure 10d illustrates the impact of WD and DT on the MGFI2 surface. When WD ranges from 0 to 0.5, the MGFI2 value increases with DT. This increase continues up to 0.65. When WD ranges from 0.5 to 1, there is a slow rise in the MGFI2 value with DT up to 0.5, followed by an increase to 1.0 and a subsequent gradual decrease to 0.7. As shown in Figure 10e, the effects of DT and MGFI1 on the MGFI2 surface are illustrated. The MGFI2 value increases to 0.65 as MGFI1 rises when DT lies between 0 and 0.5. Additionally, the MGFI2 value climbs to 1 as DT gradually increases from 0.5 to 1, and MGFI1 ranges from 0 to 1. Figure 10f illustrates the effects of WD and MGFI1 on the MGFI2 surface. When WD ranges from 0 to 0.5, and MGFI1 remains between 0 and 1, the MGFI2 value increases to 0.4. As MGFI1 transitions from 0 to 0.5 and WD shifts from 0.5 to 1, the MGFI2 value can reach 1. In summary, the aforementioned charts clearly show that the SR, PP, VH, WD and DT values can be predicted when spraying ZrO_2_ coatings. The established curves allow desirable data to be determined, and the ideal multi-properties can be obtained from this information.

### 4.9. Verification Experiments

This experiment employed a grey-based fuzzy algorithm based on a Taguchi design to determine the optimal stirring parameters. Based on the factor effects in Table 5, the optimal setting for the SNR of the multiple response (MGFI2) is A1, B3, C2, D1, E1, F3, G1 and H2. Furthermore, validation experiments have been conducted to confirm the obtained response results. The five quality attributes (SR, PP, VH, WD and DT) were normalised using grey relational analysis. The SNR for MGFI2 was then constructed using “IF–THEN” rules in the fuzzy inference engine. Optimising the spraying parameters for ceramic coatings using plasma spraying allows ideal quality attributes to be achieved, which are required for superior multi-response characteristics. These include minimal surface roughness, minimal porosity, maximum hardness levels, minimal wear depth and the desired coating thickness. Table 3 shows the results of testing for each property. Different tests reveal distinct characteristics, which cannot be compared across experimental results, thereby making it impossible to evaluate performance as a whole. For example, the lowest surface roughness was exhibited by the L5 specimen, the smallest porosity percentage was demonstrated by the L11 specimen, the highest hardness was shown by the L15 specimen, the smallest wear depth was exhibited by the L16 specimen, and the ideal coating thickness was presented by the L19 specimen. Apparently, when several characteristics are considered simultaneously, there is no discernible trend in the overall results for spraying coatings with different properties. However, by using a multi-grey-based fuzzy system to compute SNRs, the characteristics of each attribute can be effectively integrated. Optimised parameters for sprayed zirconia coatings were obtained and are shown in Table 7. The optimised parameters were validated through experimental verification. The surface roughness was seen to decrease from 8.59 μm to 7.21 μm when the optimal parameter settings were used. Porosity % was also seen to decrease from 8.59 μm to 7.84 μm, and Vickers hardness was seen to increase from 997 Hv to 1342 Hv. Furthermore, wear depth was seen to decrease from 80.47 μm to 53.26 μm^3^, and coating thickness was seen to decrease from 94.17 μm to 85.24 μm. Furthermore, the SNR increased by 1.786 dB when transitioning from the initial to the optimal settings, indicating substantial performance enhancements across various attributes of the plasma-sprayed ceramic coating. It is clear from the above that the optimal settings that produce a superior quality of coating are sufficiently robust against noise effects to result in better reproducibility.

## 5. Conclusions

The grey-based fuzzy model, as devised by Taguchi, was successfully implemented in this stud,. as an effective approach for modelling the enhancement of surface properties in plasma-sprayed ceramic coatings. An L18 orthogonal design was employed in the experiments, which were conducted with eight controllable factors. Additionally, the properties of the ceramic coatings were evaluated, including surface roughness, porosity percentage, hardness, wear depth, and deposited thickness. By introducing the SNR concept into multivariate experiments and calculating the quantitative formula derived from grey relational grades, robust optimisation of multiple quality characteristics is achieved. The grey relational grades utilise the fuzzy rule library of the inference engine to compute the weights of each grey relational coefficient, thereby synthesising an overall comprehensive grade. By applying significant factors in the analysis of variance, the significant factors identified are acceleration voltage, stand-off distance, powder feed rate, and travel speed. Collectively, these significant parameters explain approximately 78.01% of the total variance in the composite performance. Additionally, a highly dense layered structure with mechanical bonding and minor interfacial fusion adhesion was revealed by the SEM analysis of the sprayed ceramic coating. The surface roughness, porosity percentage and wear phenomena were all significantly reduced, while the hardness was enhanced. The coating showed that the thickness was even and the surface was smooth. The experimental results have confirmed that the grey-based fuzzy method based on the Taguchi design can optimise multi-property attributes simultaneously. In this study, this approach was found to effectively improve the surface properties of the substrate through plasma spraying. Furthermore, the relationship diagram between factors and characteristics can be interpreted using these characteristic results, thereby gaining valuable insights into the plasma spray coating.

## Figures and Tables

**Table 1:** The chemical compositions of spraying powdered materials [34].

Powders of Ceramic	Composite Percentage (wt.%)								
	ZrO_2_	Al_2_O_3_	SiO_2_	Y_2_O_3_	TiO_2_	Fe_2_O_3_	CaO	MgO	HfO_2_
ZrO_2_-8Y_2_O_3_	90.28	0.05	0.11	7.52	0.12	0.02	0.03	0.01	1.9

**Table 2:** Operating factors and their levels of plasma spraying coatings [34].

Symbol	Operating Factors	Level 1	Level 2	Level 3
A	Spraying layers	5	8	
B	Accelerating voltage (V)	65	70	75
C	Arc current (A)	550	600	650
D	Travel speed (mm/s)	20	25	30
E	Stand-off distance (cm)	8	10	12
F	Powder feeder rate (g/min)	20	25	30
G	Carrier gas, Ar (L/min)	5	6	7
H	Primary gas, Ar/H_2_ (L/min)	50	55	60

**Table 3:** Experimental data, including one 2-level factor and seven 3-level factors, based on orthogonal arrays with normalised values for various attributes, including surface roughness (SR), porosity percentage (PP), Vickers hardness (VH), wear depth (WD) and deposited thickness (DT) of ZrO_2_ coatings by plasma spray.

EXP	A	B	C	D	E	F	G	H	Surface Properties of Coatings					Normalisation				
									SR	PP	VH	WD	DT	SR	PP	VH	WD	DT
1	1	1	1	1	1	1	1	1	8.58	14.35	997	80.73	94.17	0.469	0.438	0.178	0.792	0.929
2	1	1	2	2	2	2	2	2	7.60	16.59	1396	115.33	44.17	0.790	0.273	0.843	0.598	0.676
3	1	1	3	3	3	3	3	3	6.97	15.42	1223	79.93	36.83	0.997	0.359	0.555	0.797	0.618
4	1	2	1	1	2	2	3	3	7.66	14.93	1122	128.8	64.50	0.770	0.395	0.387	0.523	0.836
5	1	2	2	2	3	3	1	1	10.01	9.67	1108	131.0	76.00	0.000	0.783	0.363	0.510	0.927
6	1	2	3	3	1	1	2	2	8.76	11.16	1484	156.00	84.17	0.410	0.673	0.990	0.370	0.992
7	1	3	1	2	1	3	2	3	8.57	8.15	1176	153.66	211.83	0.472	0.895	0.477	0.383	0.000
8	1	3	2	3	2	1	3	1	6.96	9.56	1014	85.56	40.93	1.000	0.791	0.207	0.765	0.650
9	1	3	3	1	3	2	1	2	9.05	10.28	958	85.13	123.00	0.315	0.738	0.113	0.768	0.702
10	2	1	1	3	3	2	2	1	7.95	20.29	1216	72.9	66.67	0.675	0.000	0.543	0.836	0.853
11	2	1	2	1	1	3	3	2	7.74	6.72	1132	71.3	81.50	0.744	1.000	0.403	0.845	0.970
12	2	1	3	2	2	1	1	3	8.29	17.71	1133	92.56	96.50	0.564	0.190	0.405	0.726	0.911
13	2	2	1	2	3	1	3	2	8.00	14.05	1346	72.03	46.33	0.659	0.460	0.760	0.841	0.693
14	2	2	2	3	1	2	1	3	7.78	8.41	890	162.66	83.32	0.731	0.875	0.000	0.333	0.985
15	2	2	3	1	2	3	2	1	8.50	15.74	1010	43.70	57.67	0.495	0.335	0.200	1.000	0.782
16	2	3	1	3	2	3	1	2	8.32	12.97	1490	84.66	129.33	0.554	0.539	1.000	0.770	0.652
17	2	3	2	1	3	1	2	3	8.52	6.99	1173	104.66	73.67	0.489	0.980	0.472	0.658	0.909
18	2	3	3	2	1	2	3	1	8.49	17.24	974	222.00	102.25	0.498	0.140	0.000	0.858	0.866

**Table 4:** Experimental SNR based on orthogonal arrays with various attributes of plasma-sprayed welds, including desired deviation, relational coefficient and relational grade, MGFI1 and MGFI2.

No. of Tests	Desired Deviation					Relational Coefficient					MFGI1	MFGI2	SNR
	ΔSR	ΔPP	ΔVH	ΔWD	ΔDT	ξSR	ξPP	ξVH	ξWD	ξDT			
1	0.531	0.562	0.822	0.208	0.078	0.485	0.471	0.378	0.707	0.868	0.441	0.655	−3.675
2	0.210	0.727	0.157	0.402	0.311	0.704	0.407	0.761	0.554	0.619	0.439	0.566	−4.944
3	0.003	0.641	0.445	0.203	0.369	0.993	0.438	0.529	0.711	0.578	0.384	0.385	−8.291
4	0.230	0.605	0.613	0.477	0.152	0.685	0.452	0.449	0.512	0.770	0.395	0.4	−7.959
5	1.000	0.217	0.637	0.490	0.062	0.333	0.697	0.440	0.505	0.893	0.710	0.674	−3.427
6	0.590	0.327	0.010	0.630	0.000	0.459	0.604	0.980	0.443	1.004	0.655	0.64	−3.876
7	0.528	0.105	0.523	0.617	0.999	0.486	0.826	0.489	0.448	0.335	0.512	0.599	−4.451
8	0.000	0.209	0.793	0.235	0.336	1.000	0.705	0.387	0.680	0.600	0.357	0.413	−7.681
9	0.685	0.262	0.887	0.232	0.304	0.422	0.656	0.361	0.683	0.625	0.583	0.589	−4.598
10	0.325	1.000	0.457	0.164	0.135	0.606	0.333	0.523	0.753	0.791	0.399	0.405	−7.851
11	0.256	0.000	0.597	0.155	0.019	0.662	1.000	0.456	0.764	0.967	0.450	0.587	−4.627
12	0.436	0.810	0.595	0.274	0.096	0.534	0.382	0.457	0.646	0.842	0.427	0.445	−7.033
13	0.341	0.540	0.240	0.159	0.294	0.595	0.481	0.676	0.759	0.632	0.477	0.483	−6.321
14	0.269	0.125	1.000	0.667	0.005	0.650	0.801	0.333	0.428	0.995	0.373	0.449	−6.955
15	0.505	0.665	0.800	0.000	0.205	0.498	0.429	0.385	1.000	0.712	0.433	0.469	−6.577
16	0.446	0.461	0.000	0.230	0.353	0.529	0.521	1.000	0.685	0.588	0.598	0.664	−3.557
17	0.511	0.020	0.528	0.342	0.080	0.494	0.962	0.486	0.594	0.865	0.497	0.622	−4.124
18	0.502	0.775	0.860	1.000	0.141	0.499	0.392	0.368	0.333	0.783	0.427	0.483	−6.321

**Table 5:** Response effect of the average value calculated by SNR for the coating.

Symbol	*A*	*B*	*C*	*D*	*E*	*F*	*G*	*H*
Level 1	−5.43	−6.07	−5.64	−5.26	−4.98	−5.45	−4.87	−5.92
Level 2	−5.93	−5.85	−5.29	−5.42	−6.29	−6.44	−5.30	−4.65
Level 3	0.00	−5.12	−6.12	−6.37	−5.77	−5.16	−6.87	−6.47
Effect	0.50	0.95	0.82	1.11	1.31	1.28	1.99	1.82
Rank	8	6	7	5	3	4	1	2

**Table 6:** The analysis of variance of SNR for ceramic coatings.

Symbol	Sum of Squares	Degree of Freedom	Mean Square	F- Value	Percent Contribution
*A*	1.107	1.0	1.107	0.464	2.24
*B*	2.960	2.0	1.480	0.620	5.99
*C*	2.051	2.0	1.025	0.430	4.15
*D*	4.320	2.0	2.160	0.905	8.74
*E*	5.198	2.0	2.599	1.089	10.52
*F*	5.414	2.0	2.707	1.134	10.95
*G*	13.194	2.0	6.597	2.764	26.70
*H*	10.403	2.0	5.201	2.179	21.05
Error	4.774	2.0	2.387	1.000	9.66
Total	49.421	17.0	2.907		100.00

**Table 7:** Comparison of propertied and SNR between initial and optimal settings for plasma-sprayed ceramic coatings.

Symbol	Initial Settings	Optimal Settings
Factor and levels	A_1_B_1_C_1_D_1_E_1_F_1_G_1_H_1_	A_1_B_3_C_2_D_1_E_1_F_3_G_1_H_2_
Surface roughness (μm)	8.58	7.21
Porosity percentage	14.35	7.84
Vicker hardness (Hv)	997	1342
Wear depth (μm)	80.75	53.26
Deposited thickness (μm)	94.1	85.24
MGFI1	0.456	0.777
MGFI2	0.661	0.807
SNR (dB)	−3.649	−1.863

## Data Availability

The original contributions presented in this study are included in the article. Further inquiries can be directed to the corresponding author.
